# Auditory cortex controls sound-driven innate defense behaviour through corticofugal projections to inferior colliculus

**DOI:** 10.1038/ncomms8224

**Published:** 2015-06-11

**Authors:** Xiaorui R. Xiong, Feixue Liang, Brian Zingg, Xu-ying Ji, Leena A. Ibrahim, Huizhong W. Tao, Li I. Zhang

**Affiliations:** 1 Zilkha Neurogenetic Institute, Keck School of Medicine, University of Southern California, Los Angeles, California 90033, USA; 2 Neuroscience Graduate Program, University of Southern California, Los Angeles, California 90033, USA; 3 Department of Physiology, School of Basic Medical Sciences, Southern Medical University, Guangzhou 510515, China; 4 Department of Cell and Neurobiology, Keck School of Medicine, University of Southern California, Los Angeles, California 90033, USA; 5 Department of Physiology and Biophysics, Keck School of Medicine, University of Southern California, Los Angeles, California 90033, USA

## Abstract

Defense against environmental threats is essential for animal survival. However, the neural circuits responsible for transforming unconditioned sensory stimuli and generating defensive behaviours remain largely unclear. Here, we show that corticofugal neurons in the auditory cortex (ACx) targeting the inferior colliculus (IC) mediate an innate, sound-induced flight behaviour. Optogenetic activation of these neurons, or their projection terminals in the IC, is sufficient for initiating flight responses, while the inhibition of these projections reduces sound-induced flight responses. Corticocollicular axons monosynaptically innervate neurons in the cortex of the IC (ICx), and optogenetic activation of the projections from the ICx to the dorsal periaqueductal gray is sufficient for provoking flight behaviours. Our results suggest that ACx can both amplify innate acoustic-motor responses and directly drive flight behaviours in the absence of sound input through corticocollicular projections to ICx. Such corticofugal control may be a general feature of innate defense circuits across sensory modalities.

## Abstract (web summary)

Defense against environmental threats is essential for survival, yet the neural circuits mediating innate defensive behaviours are not completely understood. Here the authors demonstrate that descending projections from the auditory cortex to the midbrain mediate innate, sound-evoked flight behaviour.

## Graphical abstract

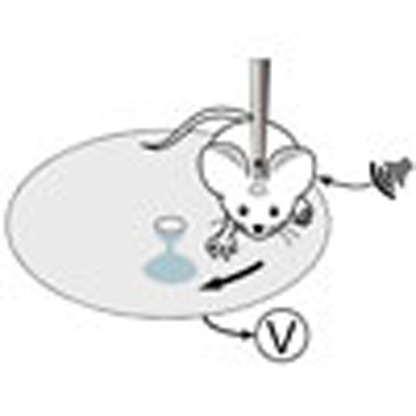

Defense against harm is a fundamental requirement for survivial. Natural threatening stimuli such as odours of predators^1^,^2^,^3^, looming shadows^4^ or warning sounds emitted by co-species^5^,^6^, can automatically invoke species-wide defensive behaviours including freezing, fleeing and fighting^7^,^8^. These innate behaviours are controlled by hard-wired, genetically preprogrammed innate defense circuits that have evolved through natural selection. Biologically insignificant or neutral sensory stimuli (that is, conditioned stimuli, CS) can acquire the ability to elicit defensive behaviours through the process of Pavlovian conditioning, when they occur in conjunction with biologically significant threats (that is, unconditioned stimuli, US).

Neural circuits for unconditioned and conditioned behaviours likely share common components, for example, the centres for organizing defensive behavioural responses such as the periaqueductal gray (PAG)^2^,^9^ and the motor pathways for producing behavioural reactions. Previous studies have largely focused on the neural mechanisms for learned defensive behaviours such as fear conditioning. For fear conditioning, it is generally believed that the sensory inputs from CS such as a tone or flash of light are processed in the cortex^10^ and are then transmitted to the amygdaloid complex, where the association between CS and US occurs^11^. Outputs from the central nucleus of the amygdala then project to the ventrolateral PAG (PAGvl) to produce fear behaviours^12^,^13^,^14^,^15^,^16^. It is surprising to note that, for many innate defense behaviours, the neural circuits responsible for producing defensive behaviours in response to the US, particularly for non-olfactory modalities are considerably less well understood^1^,^11^.

In this study, we address a general question about innate defensive behaviours: whether the sensory cortex contributes to the generation of these behaviours and if so what are the underlying neural pathways. By focusing on a sound-induced innate flight behaviour and exploiting the modern optogenetic tools with high spatial and neuronal specificity, we explored the role of auditory cortex (ACx) in the generation of the innate defensive behaviour and dissected the underlying neural pathway. Our results reveal that ACx can drive the innate defensive behaviour via the corticofugal projections to the cortex of the inferior colliculus (IC), which then connects to the PAG to initiate the flight response. Our study elucidates a previously unrecognized role of corticofugal projections in mediating innate sensory-motor responses, which may be general across sensory modalities.

## Results

### A sound-induced innate defensive behaviour

**Figure 1:**
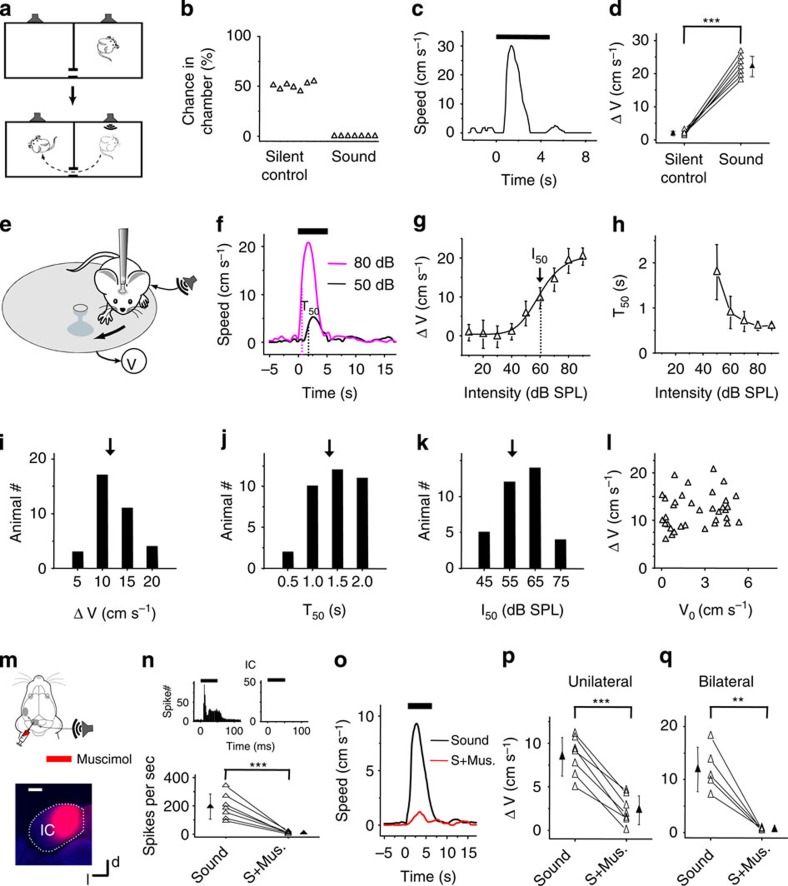
IC-dependent auditory-induced innate flight behaviour. (**a**) Schematic graph for the flight behaviour test. When sound was played in the chamber the mouse stayed in, it quickly ran to the other chamber. (**b**) Percentage chance of animal staying in the chamber playing sound or silent sound 10 s after the trigger signal. *N*=7 animals. (**c**) Fleeing speed trace for an example animal. Thick bar marks the sound duration. (**d**) Averaged speed of all the tested animals (*n*=7). ***P<0.001, paired *t*-test. (**e**) Head-fixed preparation. Sound was delivered to one ear (with the other ear plugged). Running speed (*V*) was monitored in real time. (**f**) Average speed of an example animal (50 trials, inter-stimulus interval=30 s) in response to noise (marked by the thick bar) at two intensity levels. Dash vertical line marks T_50_. (**g**) Peak induced speed (average of 30 trials or more) versus noise intensity plotted for the same animal in (**f**). Dash vertical line marks I_50_. (**h**) T_50_ versus noise intensity for the same animal. (**i**) Distribution of average peak induced speeds (*n*=35 animals). Arrow marks the mean value. (**j**) Distribution of T_50_. (**k**) Distribution of I_50_. (**l**) Peak induced versus baseline speed. (**m**) Top, muscimol was injected into the IC contralateral to the sound source. Bottom, fluorescence image of a representative IC slice showing the spread of muscimol. Scale bar, 500 μm. d, dorsal; l, lateral. (**n**) Top, peri-stimulus spike-time histograms (PSTHs, 100 trials) for responses of an IC neuron to noise before (left) and after (right) muscimol injections. Bottom, average spike rates evoked by noise (80 dB SPL) before and after (S+ Mus.) muscimol injections for 7 IC cells in seven animals. Recording depths ranged from 250–800 μm. ***P<0.001, paired *t*-test. (**o**) Speed trace of an example animal before (black) and after (red) muscimol injections into the IC. (**p**) Summary of the peak induced speed before and after silencing the contralateral IC. ***P<0.001, paired *t*-test. *N*=7 animals. (**q**) Summary of the peak induced speed before and after silencing IC bilaterally. **P<0.01, paired *t*-test. *N*=5 animals. All error bars represent s.d.

By examining acoustically induced behavioural reactions, we observed that a moderately loud sound could reliably invoke flight responses in naïve mice. As shown in Fig. 1a–d, during free exploration in a chamber, a broadband noise (1–64 kHz; 80 dB sound pressure level (SPL), 5-s duration) delivered from a hidden speaker in the chamber resulted in a flight behaviour away from the sound to another connected chamber. The animal typically remained on the far side of the second chamber for the remainder of the noise. No obvious freezing behaviour was observed before the flight or during the noise presentation. Such innate defensive behaviour (that is, fleeing from potential threats) was observed in all of the mice tested (*n*=7).

To study the neural circuits for this sound-induced flight behaviour, we developed a head-fixed preparation^17^,^18^,^19^ so that the behavioural response could be easily quantified. Head-fixed mice were habituated to rest or run on a flat turntable and the running speed was recorded in real time (Fig. 1e). A 5-s noise presented at 50 dB SPL on one side of the animal (with the contralateral ear plugged) induced running ∼1 s after the onset of noise, as shown by the increase in turntable speed (Fig. 1f, black). Noise presented at a higher intensity (80 dB SPL) resulted in faster running speeds and a more rapid response initiation (Fig. 1f, magenta). The sound-induced running behaviour was further confirmed by examining recorded video images. By varying the intensity of the noise in a randomized order, we determined the relationship between the peak sound-induced running speed and the stimulus intensity (Fig. 1g), which could be modelled using a hyperbolic ratio function (see Methods). We defined the intensity at which the speed reached the half-maximum value as I_50_ (Fig. 1g). We also measured T_50_, defined as the time when the sound-induced running speed reached the half-maximum value, relative to the onset of noise (Fig. 1f). T_50_ decreased with increases in the stimulus intensity (Fig. 1h), indicating that increasing noise intensity resulted in faster initiation of the flight response. Such noise-induced flight behaviour was reliably observed in a total of 35 head-fixed mice, although there was substantial variability in the induced running speed (ΔV), T_50_ and I_50_ across mice (Fig. 1i–k, respectively). There was no correlation between sound-induced speed and the baseline speed (correlation coefficient=0.19, Fig. 1l), indicating that the sound-induced running was relatively independent of the initial status of the animal. When both ears were left open, noise at 80 dB SPL produced similar running speeds as in the condition of unilateral plugging (Supplementary Fig. 1a). The behavioural response under our experimental condition (with an inter-stimulus interval of 30 s) was relatively stable over the 100-min duration, without obvious adaptation, but reduced responses were observed over time when a shorter inter-stimulus interval was applied (Supplementary Fig. 1b). Further, a 5-kHz pure tone stimulus presented at 80 dB SPL evoked similar flight responses as noise (Supplementary Fig. 1c,d), suggesting that flight responses may be generally induced by loud sounds when there are escape routes.

### IC mediates the sound-triggered flight response

Previous studies have implied that the IC may be involved in acoustically induced defensive behaviours, since electric or chemical stimulation of the IC can result in defense-like responses^7^. We therefore examined the involvement of the IC in the sound-induced flight behaviour by silencing the IC with local microinjections of muscimol (see Methods). Using fluorescent muscimol, we confirmed that muscimol was largely restricted to the IC structure after the injection (Fig. 1m). The effectiveness of silencing was confirmed by loose-patch recordings from neurons across different depths of the IC (Fig. 1n). Silencing the IC contralateral to the stimulated ear largely reduced noise-induced running (Fig. 1o,p). The small residual response (Fig. 1p) might be attributed to the incomplete silencing of IC, for example, the auditory pathways through the other (ipsilateral) IC were intact. Indeed, when we silenced the IC bilaterally, the running response was almost completely abolished (Fig. 1q). These data suggest that the sound-induced flight behaviour is initiated at or above the level of the IC.

### ACx amplifies and directly drives defensive flight behaviour

**Figure 2:**
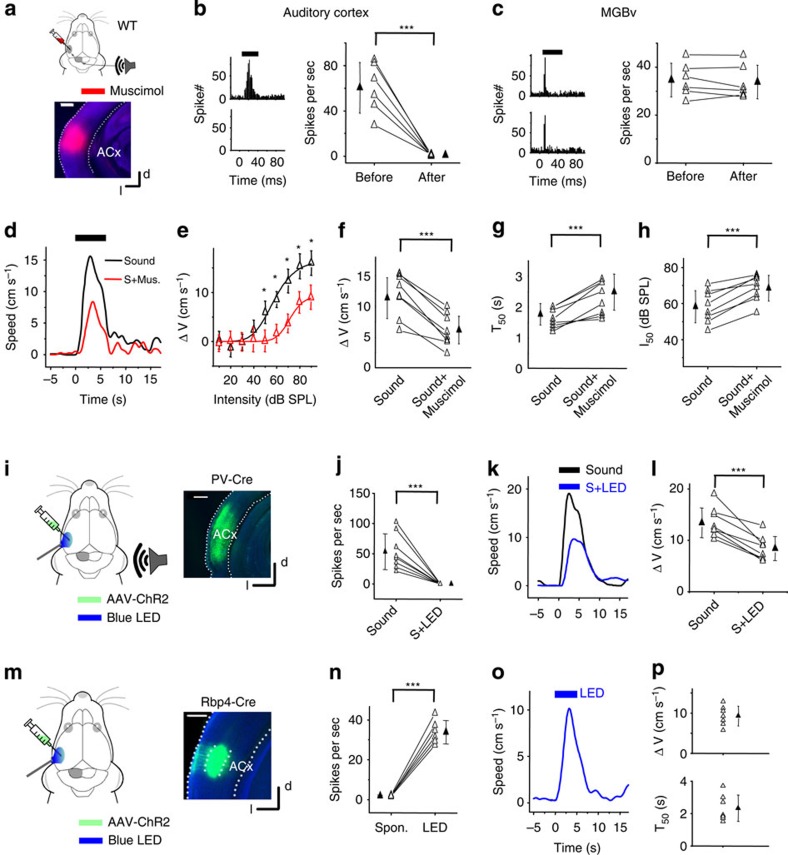
ACx mediates noise-induced flight behaviours. (**a**) Top, experimental condition. Bottom, image showing spread of fluorescent muscimol. (**b**) Left, PSTHs (100 trials) for a cortical L5 neuron’s responses to noise (80 dB SPL) before (upper) and after (lower) muscimol (Mus.) injections into the ACx. Right, summary of average spike rates evoked by noise. ***P<0.001, paired *t*- test. *N*=6 cells in six animals. (**c**) Left, PSTHs (100 trials) for an MGBv neuron’s responses to noise before (upper) and after (lower) muscimol injections into the ACx. Right, summary of average evoked spike rates for six MGBv cells. ***P<0.001, paired *t*-test. (**d**) Speed trace for an example animal before (black) and after (red) muscimol injections into the ACx. (**e**) Peak induced speed versus noise intensity for the same animal. *P<0.05, two-sample *t*-test. (**f**) Summary of peak induced speed before and after silencing the ACx with muscimol. ***P<0.001, paired *t*-test. *N*=8 animals. (**g**) Summary of T_50_. ***P<0.001, paired *t*-test. *N*=8. (**h**) Summary of I_50_. ***P<0.001, paired *t*-test. *N*=8. (**i**) Top, experimental condition. Right, image showing ChR2 expression in the ACx. (**j**) Average spike rates of L5 neurons to noise (60 dB SPL) without and with LED illumination. ***P<0.001, paired *t*-test. *N*=8 cells in three animals. (**k**) Speed trace of an example animal in response to noise (black) and noise plus LED (blue). (**l**) Average peak induced speed without and with LED illumination. ***P<0.001, paired *t*-test. *N*=8 animals. (**m**) Top: experimental condition. Right, image showing fluorescence-labelled L5 neurons primarily in the primary ACx (A1). (**n**) Summary of spontaneous (Spon.) firing rates and average multiunit spike rates evoked by LED stimulation (50 ms pulse) in L5 of A1. ***P<0.001, one sample *t*-test. *N*=7 sites in seven animals. (**o**) Speed trace (average of 35 trials) of an animal in response to LED stimulation. (**p**) Top, summary of peak induced speed to LED stimulation. *N*=7 animals. Bottom, summary of T_50_. Scale bar, 500 μm. All error bars represent s.d.

For any sound-induced behaviour, a straightforward question to ask is whether the ACx is involved. The ACx processes ascending acoustic information relayed from the IC (ref. 20) and may contribute to the evaluation of threat signals. To silence the ACx, we locally injected muscimol, which diffused mainly throughout the primary auditory cortical region (A1), leaving subcortical structures and other cortical areas (for example, motor cortex) unaffected (Fig. 2a). Loose-patch recordings confirmed that sound-evoked spike responses in the A1 were effectively eliminated (Fig. 2b). On the other hand, the responses in the auditory thalamus (the ventral part of the medial geniculate nucleus (MGBv)) remained intact (Fig. 2c), indicating that the ascending auditory pathway from the IC to the MGBv and then to the ACx was not affected by cortical muscimol injections. Silencing the ACx contralateral to the sound source markedly attenuated, but did not abolish, the sound-induced flight response (Fig. 2d), resulting in a rightward shift of the speed-intensity curve and a reduction of the maximum induced speed (Fig. 2e). On average, ΔV was reduced by 47% after silencing the ACx (Fig. 2f). Moreover, both T_50_ and I_50_ increased (Fig. 2g,h). Silencing the ACx bilaterally resulted in a similar reduction in ΔV (data not shown). In control experiments where saline was injected into the ACx, or muscimol into the visual cortex, no significant change in the behavioural response was observed (Supplementary Fig. 2).

We also applied another method of cortical silencing, by optogenetically activating parvalbumin (PV) positive inhibitory neurons^21^,^22^. We injected an adeno-associated viral vector encoding double-floxed inverted channelrhodopsin 2 (ChR2; ref. 23) fused with enhanced yellow fluorescent protein (EYFP; AAV-DIO-ChR2-EYFP) into the ACx of PV-Cre mice (Fig. 2i). PV neurons were optically activated by blue light-emitting diode (LED) light (470 nm) applied to the cortical surface through an optic fibre. Loose-patch recordings of excitatory cells in deep layers of A1 (see Methods) confirmed that their responses could be silenced by a train of pulses of blue LED illumination (10 ms pulse duration, 10 Hz; Fig. 2j). We interleaved noise stimulation with and without blue LED illumination, and found that LED illumination markedly reduced noise-induced running speeds (Fig. 2k). On average, the peak induced running speed was reduced by 40% (Fig. 2l), comparable to the effect of muscimol silencing (Fig. 2f). Together, these results suggest that subcortical structures are partially responsible for producing the behavioural response and that ACx-mediated processes amplify and accelerate the response. In addition, these results raise the interesting possibility that ACx can directly drive the defensive behaviour.

Since layer 6 of the primary sensory cortex has been shown to play a primary role in controlling the gain of thalamocortical inputs^17^ and layer 2/3 in sending information to higher cortical areas for sensory processing^24^, we suspected that layer 5 (L5), another cortical output layer, might play a prominent role in mediating the observed behaviour^25^. To test this hypothesis, we injected the AAV-DIO-ChR2-EYFP into the A1 region of a L5-specific Cre driver mouse line, Rpb4-Cre^26^, to optically activate only this output layer (Fig. 2m). The EYFP-labelled L5 neurons exhibited a long apical dendrite ascending towards layer 1 (Fig. 2m), consistent with previous morphological studies of L5 pyramidal cells^27^,^28^. We applied blue LED light to the cortical surface, with the tip of the optic fibre covered by dark stained agar to minimize the leakage of LED light (see Methods). LED illumination induced spiking responses of L5 neurons (Fig. 2n and Supplementary Fig. 3a,b). Surprisingly, the optical activation of L5 neurons induced running (Fig. 2o,p). This behavioural response depended on the LED light intensity, and was not evoked at low LED powers (Supplementary Fig. 3c,d). In addition, the running behaviour could not be attributed to visual effects, as control mice injected with AAV-GFP did not respond to the same LED illumination (Supplementary Fig. 3e,f). In view of the above results, we reasoned that a common neural pathway through the same subcortical sensory structures might enable the ACx to amplify the sound-induced flight behaviour and to drive behavioural output in the absence of sensory input.

### Involvement of corticofugal projections to the cortex of IC

**Figure 3:**
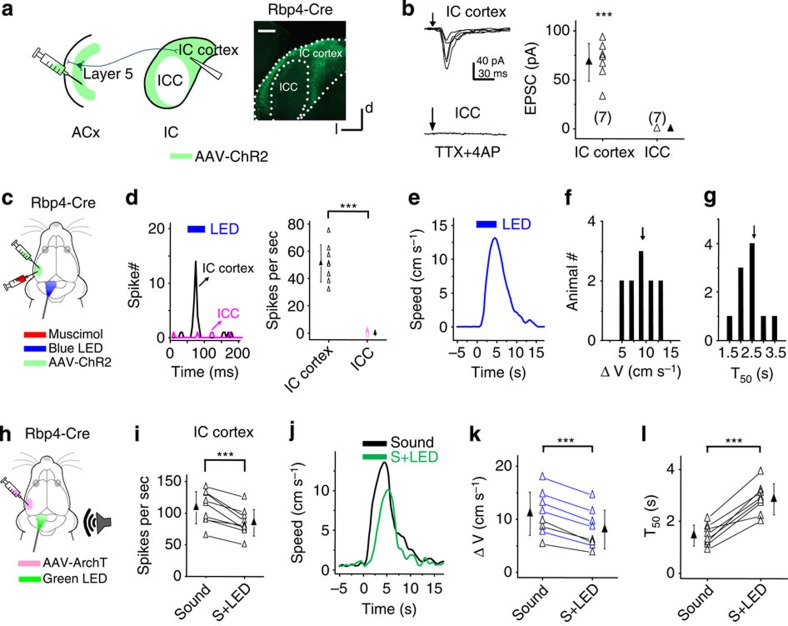
Involvement of corticocollicular projections. (**a**) Left: experimental condition. Right, image showing distribution of corticofugal axons in the IC. Scale bar, 500 μm. (**b**) Left, EPSCs evoked by blue LED illumination (10 ms pulse) in an example ICx and ICC neuron, recorded under −70 mV. Arrow marks the onset of LED illumination. Right, summary of average EPSC amplitudes to LED stimulation. ***, different from zero, *P*<0.001, *t*-test. *N*=7 cells in each group. (**c**) In a head-fixed animal, blue LED light was applied to the IC surface to activate corticocollicular axon terminals with muscimol injected into the ACx on the same side. (**d**) Left, PSTHs (100 trials) of multiunit spike responses to LED illumination (blue bar) in an example ICx and ICC recording. Right, summary of average evoked spike rates. ***P<0.001, *t*-test. *N*=8 sites for each group. (**e**) Speed trace of an example animal in response to the LED illumination on the IC surface. (**f**) Distribution of peak induced speeds to LED stimulation (*n*=10 animals). (**g**) Distribution of T_50_. (**h**) AAV-ArchT was injected into the ACx of a Rbp4-Cre mouse. Six to eight weeks later green LED light was applied to the IC on the same side and contralateral to the sound source. (**i**) Average spike rates of ICx neurons evoked by noise without and with green LED illumination. ***P<0.001, paired *t*-test. *N*=9 cells in five animals. (**j**) Speed trace of an example animal in response to noise (black) and noise plus green LED illumination (green). (**k**) Summary of peak induced speeds without and with green LED illumination. ***P<0.001, paired *t*-test. *N*=8 animals. Blue symbol labels an individual animal for which there was a significant reduction in speed (*P*<0.05, *t*-test). (**l**) Summary of T_50_. ***P<0.001, paired *t*-test. All error bars represent s.d.

Since ACx connects to the IC through corticofugal projections originating from L5 (refs 29, 30), we directly examined whether L5 neurons can drive the flight behaviour via the corticocollicular projections. In the Rbp4-Cre mice injected with AAV-DIO-ChR2 in the A1 region, we found that the EYFP-expressing corticocollicular axons in the IC were mainly distributed in the IC cortex (ICx), the shell region surrounding the central nucleus (that is, ICC; Fig. 3a). To test whether the corticocollicular axons innervate IC neurons, we performed whole-cell voltage-clamp recordings in slice preparations, and stimulated corticocollicular axon terminals by illuminating blue LED light onto the entire IC region (see Methods). The slices were bathed in 1 μM TTX (tetrodotoxin) and 1 mM 4-aminopyridine so that polysynaptic responses were blocked^31^. We found that a 10-ms pulse of LED light induced robust excitatory postsynaptic currents (EPSCs) in neurons in the ICx (Fig. 3b), demonstrating that the corticocollicular axons indeed monosynaptically innervate these cells. On the other hand, no EPSCs were observed in ICC neurons recorded in the same slices, indicating that the probability of ICC neurons being innervated by corticocollicular axons is much lower compared with ICx neurons, consistent with the sparseness of corticocollicular axons in the ICC region (Fig. 3a).

We next examined whether the local activation of corticocollicular axon terminals in the IC could directly drive the flight behaviour. In head-fixed Rbp4-Cre animals injected with AAV-DIO-ChR2, we applied a train of LED light pulses (10 ms duration, 10 Hz) onto the IC surface for 5 s, while the ACx on the same side had been silenced with muscimol to prevent potential antidromic stimulation of L5 neurons (Fig. 3c). Under this condition, the LED illumination increased spiking activity of neurons in the ICx, but not in the ICC (Fig. 3d). Optogenetic activation of the corticocollicular axon terminals in the IC evoked running (Fig. 3e), with similar running speeds as those induced by noise (Fig. 3f), but with longer latencies (Fig. 3g, T_50_, 2.62±0.56 s versus 1.44±0.45 s, *P*<0.01, *t*-test). The latter could be due to a longer time needed for activating a sufficient number of neurons in the ICx under our current optical stimulation conditions as compared with natural sound stimulation. Together, our data suggest that the activation of corticocollicular projections is sufficient for driving the flight behaviour.

To further confirm that corticocollicular projections are involved in the sound-induced flight behaviour, we injected AAV-flexed-ArchT-GFP^32^ into the ACx of Rpb4-Cre mice to suppress spiking activity of L5 neurons (Fig. 3h). In cortical slices, green LED light illumination (530 nm, 1 s) caused a large hyperpolarization of the membrane potential in fluorescence-labelled L5 pyramidal neurons (−33±8 mV, *n*=10 cells), confirming the effectiveness of ArchT expression. In head-fixed animals, green LED light illumination onto the auditory cortical surface reduced the sound-induced running speed by 40% (Supplementary Fig. 4a–d). Consistently, illumination on the IC surface suppressed sound-evoked spike responses of ICx neurons (Fig. 3h,i) and reduced the sound-induced behavioural response by 27% (Fig. 3j–l). Possibly due to less efficient inhibition at axon terminals, the level of reduction was smaller compared with the illumination on the cortical surface. The effects of activation and suppression of the corticocollicular projections to the IC with various methods (Supplementary Fig. 5) together strongly indicate their involvement in the sound-induced flight behaviour.

### ICx drives the flight behaviour

**Figure 4:**
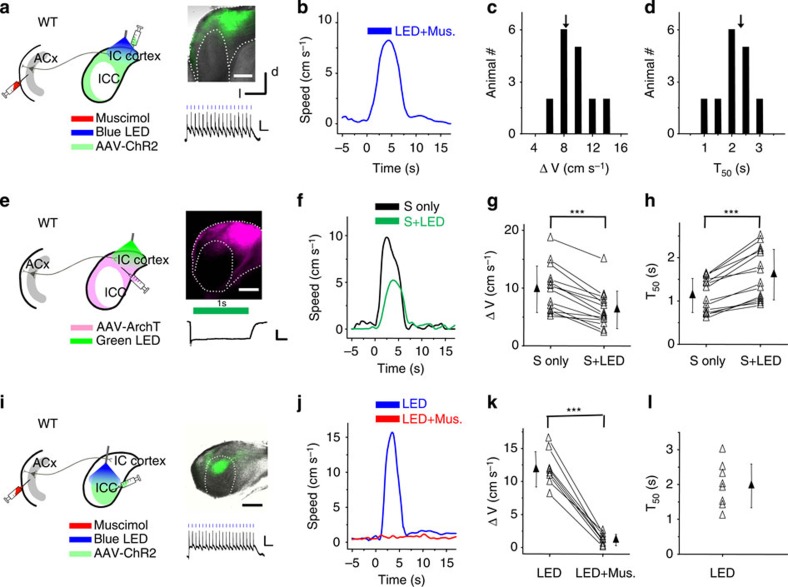
ICx mediates the noise-induced fleeing behaviour. (**a**) Left, AAV-ChR2-EYFP was injected into the dorsal cortex of IC. Blue LED light was applied to the IC surface while ACx was silenced with muscimol (Mus.). Right top, fluorescence (green) image superimposed on the bright field image of an IC slice injected with AAV-ChR2. Right bottom, representative membrane potential response of a ICx neuron to pulses of LED illumination. Each blue line indicates one pulse. Scale bars, 25 mV, 200 ms. (**b**) Speed trace of an example animal to blue LED illumination on the IC surface. (**c**) Distribution of average peak induced speeds to LED illumination. *N*=17 animals. (**d**) Distribution of T_50_. (**e**) AAV-ArchT was injected into the dorsal cortex of IC. Green LED light was applied to the IC surface. Right top, fluorescence (pink) image of an IC slice injected with AAV-ArchT. Right bottom, representative membrane potential response of an ICx neuron to green LED illumination. Scale bar, 15 mV, 250 ms. (**f**) Speed trace of an example animal to noise (black) and noise plus green LED illumination (green). (**g**) Summary of noise-induced speed without and with LED illumination. ***P<0.001, paired *t*-test. *N*=14 test sessions from seven animals. (**h**) Summary of T_50_. ***P<0.001, Wilcoxon signed-rank test. (**i**) AAV-ChR2 was injected into the ICC. Blue LED was applied to the IC surface. The ACx later was silenced with muscimol. Right top, fluorescence (green) image of an IC slice injected with AAV-ChR2. Note that the fluorescence in the ICx was attributed to labelled axons from ICC. Right bottom, representative membrane potential response of an ICC neuron to blue LED pulses. Scale bars, 25 mV, 200 ms. (**j**) Speed trace of an example animal to blue LED illumination before (blue) and after (red) muscimol injections into the ACx. (**k**) Summary of average peak induced speeds to LED illumination before and after muscimol injections into the ACx. ***P<0.001, paired *t*-test. *N*=10 animals. (**l**) Summary of T_50_. Scale bar, 500 μm. All error bars represent s.d.

The IC structure is anatomically separated into the ICC and the shell region (ICx). The latter has been implicated in multisensory interactions^29^,^33^. We have now shown that the activation of corticocollicular axons can drive flight behaviour, and that ICx neurons are directly innervated by corticocollicular axons (Fig. 3a,b). To confirm that the ICx is a sensory hub mediating the sound-induced behaviour, we injected an AAV-encoding nonfloxed ChR2 driven by a CaMKII promoter into the dorsal cortex of the IC (Fig. 4a). ICC neurons were largely uninfected (Fig. 4a, right panel). Whole-cell recordings in IC slices indicated that blue LED light (10 ms, 10 Hz) could reliably induce spiking of infected ICx neurons (*n*=10 cells) (Fig. 4a, right bottom), while no excitatory currents were observed in ICC neurons (*n*=10 cells, data not shown). These results demonstrate the specificity of ChR2 expression in the ICx.

In head-fixed animals, optogenetic activation of ICx neurons (and ACx silenced with muscimol) induced running (Fig. 4b–d). In a second set of experiments, we suppressed spiking activity of ICx neurons by injecting AAV–CAG-ArchT-GFP into the ICx (Fig. 4e). Green LED light illumination of ArchT-expressing neurons resulted in a large membrane hyperpolarization of 35±9 mV in these neurons (*n*=10 cells) (Fig. 4e, right bottom). In head-fixed animals, green LED illumination (5 s duration, 10 Hz) of the IC surface largely reduced the sound-evoked flight response (Fig. 4f). On average, there was a 37% reduction in the peak induced running speed (Fig. 4g), with the latency of the behavioural response prolonged (Fig. 4h). It should be noted that it is usually difficult to achieve a complete inactivation with ArchT expression. Nonetheless, the above data further suggest that the ICx is required for the sound-induced flight behaviour.

We also examined how ICC might contribute to the flight behaviour. We injected the AAV-encoding nonfloxed ChR2 into the ICC (Fig. 4i). Recordings from infected ICC neurons in slice preparations confirmed that they could be activated by blue LED light (*n*=8 cells; Fig. 4i, right bottom). Notably, LED illumination of the IC and thus the activation of ICC neurons evoked a running response similar to that induced by noise except for a longer response delay (Fig. 4j–l). However, after muscimol was injected into the ACx, this response disappeared (Fig. 4j,k). Since the MGBv relays ascending auditory input from the ICC to the ACx, we further examined the effect of silencing the MGB with muscimol on the response induced by the optical activation of the ICC. The behavioural response was largely reduced (Supplementary Fig. 6), similar to the effect of silencing the ACx. Together, these results suggest that ICC by itself does not directly mediate the flight behaviour. It mainly relays ascending auditory input into the ACx via the MGB, and the ACx in turn activates the ICx via the corticocollicular projections to modulate the running behaviour.

### ICx drives the midbrain defense system

**Figure 5:**
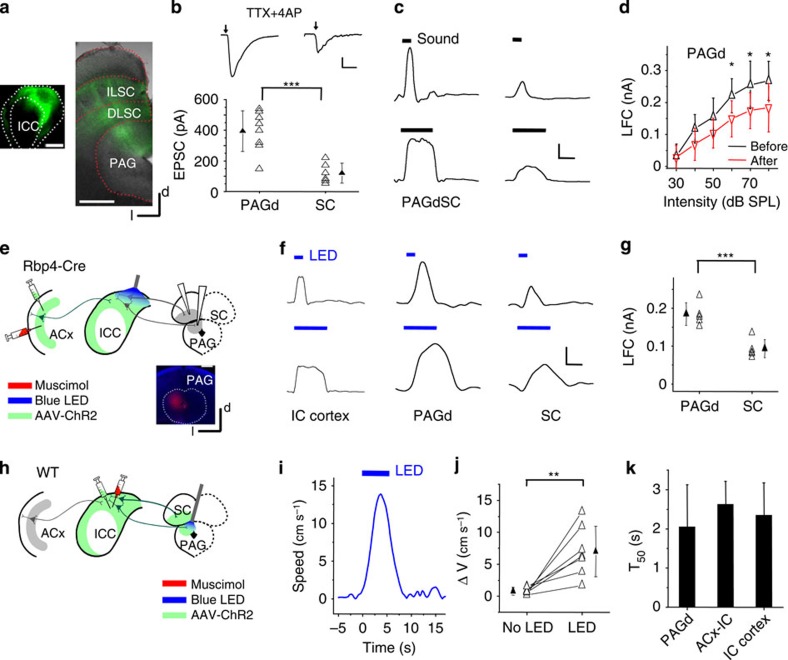
ICx drives the midbrain defense system. (**a**) Confocal image of fluorescence-labelled axons from the ICx in the intermediate layer of SC (ILSC), deep layer of SC (DLSC) and dorsal medial PAG ventral to the SC. Left inset, injection site in the ICx. (**b**) Top, average monosynaptic EPSCs recorded in a representative PAGd (left) and SC (right) neuron to LED illumination of ChR2-expressing IC axons. Scale: 50 pA, 30 ms. Bottom, summary of average monosynaptic EPSC amplitudes in PAGd (*n*=8) and SC (*n*=6) neurons in slice recordings. ***P<0.001, two-sample *t*-test. (**c**) LFCs recorded in the PAGd and ventral SC in response to noise of different durations in an example animal. Scale: 0.1 nA, 100 ms. (**d**) Average amplitudes of evoked LFCs at different noise intensities recorded in the PAGd before and after muscimol injections into the ACx. *P<0.05, paired *t*-test. *N*=4 animals. (**e**) AAV-ChR2 was injected into the ACx of Rpb4-Cre mice. Blue LED was applied to the IC surface while ACx was silenced with muscimol. Recordings were made in the PAGd and ventral SC. Bottom, image showing injected fluorescent dextran after recording in the PAGd. (**f**) Sample LFCs evoked by LED pulses of different durations recorded in the ICx, PAGd and ventral SC, for an example animal. Scale: 0.1 nA, 100 ms. (**g**) Summary of average LFC amplitudes recorded in the PAGd (*n*=5 sites from 2 animals) and ventral SC (*n*=5 sites from 3 animals). ***P<0.001, two-sample *t*-test. (**h**) AAV-ChR2 was injected into the ICx. Blue LED was applied to the PAGd, while muscimol was injected into the ICx. (**i**) Speed trace of an example animal to blue LED illumination of the PAGd. (**j**) Summary of baseline running speed and average peak induced speed to LED illumination. **P<0.01, paired *t*-test. *N*=7 test sessions from 4 animals. (**k**) Summary of average T_50_ to stimulation of the PAGd, corticofugal axons in the IC and ICx neurons. No difference was detected. *P*>0.05, one-way analysis of variance test. All scale bar: 500 μm. All error bars represent s.d.

What are the downstream targets of the ICx that produce the flight behaviour? ICx neurons primarily project their axons to the MGB^29^, intermediate and deep layers of the superior colliculus (SC) and the dorsal–lateral part of the PAG (PAGd) ventral to the SC (Fig. 5a). The PAG region ventral to the IC was not innervated by the projections from the IC (ref. 29). Previously the SC and PAG have been implicated in the midbrain defense system, as electrically or chemically stimulating these nuclei could produce a variety of defense-like behaviours^7^. It is therefore possible that ICx drives the flight behaviour by activating the SC and PAGd. Supporting this notion, in midbrain slices from mice injected with AAV-ChR2 in the ICx, blue LED illumination elicited large monosynaptic EPSCs in PAGd neurons, with their amplitudes consistently reaching hundreds of picoamperes or above (Fig. 5b). Neurons in the intermediate and deep layers of SC also received direct excitatory input from the ICx, although the input was much weaker compared with that to the PAGd (Fig. 5b). In addition, *in vivo* recording of local field currents (LFCs) in the PAGd and deep layers of SC indicated that these nuclei received sound-driven synaptic inputs (Fig. 5c). The input to the PAGd was much stronger than that to the SC (Fig. 5c), consistent with the *ex vivo* result (Fig. 5b). In addition, the amplitude of high-intensity noise evoked LFC in the PAGd was found to be significantly reduced after ACx was silenced by muscimol (Fig. 5d), indicating that ACx amplified the sound-evoked responses in the PAGd. This is consistent with the result that ACx activity amplified the sound-evoked flight behaviour (Fig. 2f,l).

To address whether ACx and ICx mediate the flight behaviour via the PAGd and SC, we first examined their responses to the activation of corticocollicular axons. In Rbp4-Cre mice injected with AAV-DIO-ChR2 in the ACx, we applied blue LED illumination onto the IC surface, with the ACx silenced with muscimol (Fig. 5e). The optogenetic activation of corticocollicular axon terminals in the IC produced LFC responses in the ICx, PAGd and deep layers of SC (Fig. 5f), confirming the L5→ICx→PAGd/SC pathway. Again, the evoked LFC responses were stronger in the PAGd than in the SC (Fig. 5g). In another set of experiments, we injected AAV-ChR2 into the ICx. We then implanted an optic fibre in the midbrain rostral to the IC to illuminate the PAGd area ventral to the SC, thus to specifically activate axon terminals from the ICx in this region (Fig. 5h). Muscimol was injected to silence the ICx region expressing ChR2 (Fig. 5h). Notably, LED illumination alone evoked running (Fig. 5i,j), and the T_50_ of the average response trace appeared similar as that under the optogenetic activation of corticocollicullar axons in the IC and of ICx neurons (Fig. 5k). Although it would be difficult to separately illuminate the SC without affecting the PAGd (see Fig. 5a), it is likely that the ICx to SC projection can also contribute to the flight behaviour, as anatomical studies have indicated that deep layers of SC project to the PAGd^34^,^35^. Together, our data suggest that ICx directly drives the midbrain defense system, in particular the PAGd, to produce the sound-triggered flight behaviour.

### Corticofugal projections to the SC are unlikely involved

**Figure 6:**
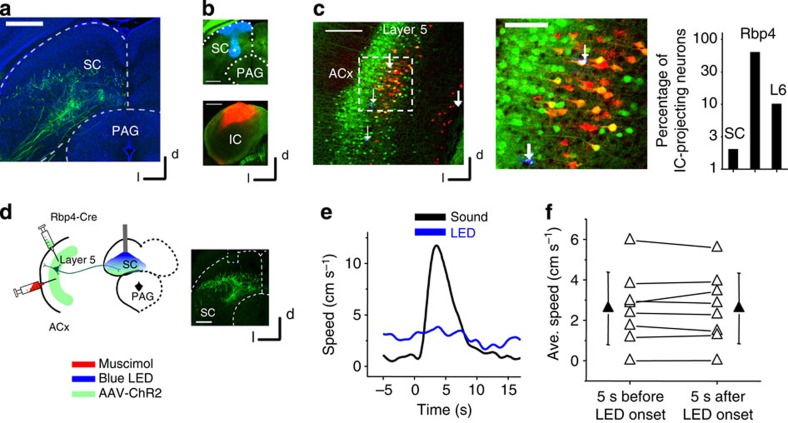
Corticofugal projections to SC unlikely contribute to the noise-induced flight behaviour. (**a**) Labelling of corticofugal axons from L5 of the ACx to the intermediate and deep layers of SC. Scale bar, 500 μm. (**b**) Double injection of two different retrograde tracers into deep layers of SC (blue, top) and the ICx (red, bottom) of a Rbp4-Cre GFP mouse. Scale bar, 500 μm. Note that the blue staining in the superficial layer of SC was due to leakage (injection site marked by *). (**c**) Left, retrograde labelling of SC- and IC-projecting neurons in the ACx. Red neurons project to the IC, blue neurons (marked by white arrows) to the SC. SC-projecting neurons are scarce (only four neurons in the view field). Scale bar, 250 μm. Middle, a blow-up image of the boxed area on the left. One out of four SC-projecting neurons also projects to the IC (labelled by white colour). Scale bar, 100 μm. Right, percentage overlap between retrogradely labelled IC-projecting cortical neurons with retrogradely labelled SC-projecting cortical neurons, genetically labelled L5 cells in the Rpb4-Cre mouse and with layer 6 neurons. (**d**) Injection of AAV-ChR2-EYFP-abelled L5 axon terminals in SC deep layers. Blue LED was applied to the SC, while the ACx was silenced with muscimol. Right, fluorescence image showing the ACx-SC axon terminals and optic fibre placement. Scale bar, 500 μm. (**e**) Speed trace of an example animal in response to blue LED illumination of the SC (blue) and to 80 dB SPL noise (black). (**f**) Speeds averaged (Avg.) within a 5-s window before and after the onset of LED illumination in SC deep layers. *N*=8 animals. No difference was detected. *P*=0.97, two sided paired *t*-test. All error bars represent s.d.

Anatomical evidence suggests direct corticofugal projections from the ACx to the SC^29^,^30^. As electric or chemical stimulation of the SC also results in some types of defense-like behaviour^7^, we further examined the possible involvements of corticofugal projections to the SC in the observed behaviour. In Rbp4-Cre mice with AAV-DIO-ChR2 injected in the ACx, we only observed weak fluorescence in the intermediate and deep layers of SC (Fig. 6a), suggesting that only a small population of the auditory cortical neurons project to the SC. Double retrograde labelling (see Methods, Fig. 6b) revealed that there was a limited overlap between L5 corticofugal neurons projecting to the IC and those to the SC (Fig. 6c). Overall, only ∼2% of IC-projecting cortical neurons also projected to the SC. Moreover, ∼66% of IC-projecting neurons were also Cre positive and a small fraction (about 10%) of IC-projecting neurons came from layer 6 (Fig. 6c). The number of labelled IC-projecting neurons was estimated to be ∼ 20 times that of SC-projecting neurons. IC-projecting neurons were found to be more enriched in L5 of primary as opposed to secondary auditory areas (Supplementary Fig. 7), consistent with previous anatomical studies^36^ and recent neural tracing results, for example, in the Allen database (connectivity.brain-map.org) and the mouse connectome project (www.mouseconnectome.org). Local activation of corticofugal axons in the SC failed to invoke running (Fig. 6d–f), which could be explained by the relatively sparse ACx-SC projection. Together, the corticofugal projection to the SC unlikely contributes directly to the sound-triggered flight response. It is possible that corticofugal projections to different subcortical targets play distinct roles in various brain functions^25^.

## Discussion

**Figure 7:**
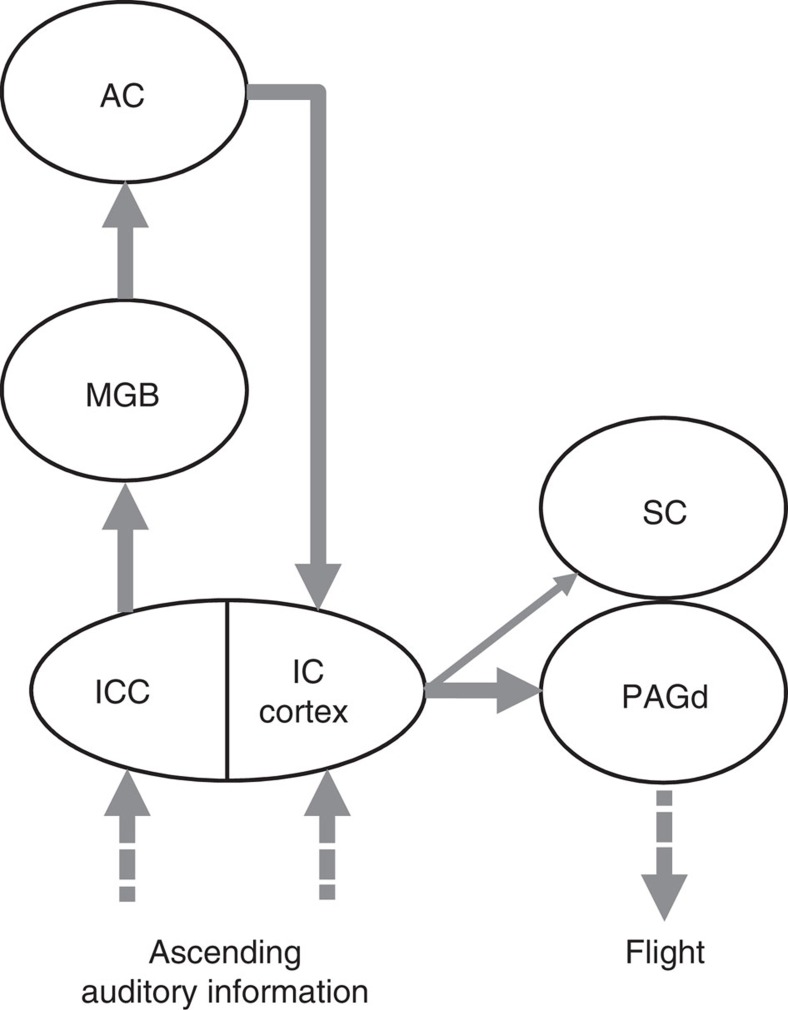
Schematic illustration of the neural pathways underlying the sound-evoked flight behaviour. Note that besides the feedback projection from the ACx, the ICx in the midbrain also receives direct ascending auditory inputs from the lower brainstem nuclei.

Flight behaviours invoked by threatening sensory stimuli have been observed in various species^8^. However, the sensory cortical control of innate defense behaviours induced by unconditioned sensory inputs and the underlying neural pathways remains unclear. In this study, we demonstrated that ACx can drive the ICx via corticofugal projections and that the ICx connects to the midbrain defense system to generate innate sound-induced flight behaviours. Our results elucidate an innate defense circuit that links the auditory sensory system and motor outputs, by which the ACx mediates sound-induced defense behaviours (Fig. 7).

The defense circuit identified in this study could be distinguished from that for other types of acoustically induced defensive responses, such as the acoustic startle response (ASR) and freezing. ASR is triggered by extremely loud sounds (>80 dB SPL), has a fast onset (with a delay that can be as short as 10 ms) and is only transient (lasting ∼100 ms; refs 37, 38). ASR is mediated by brainstem auditory nuclei below the level of the IC, for example, dorsal and ventral cochlear nucleus as well as the caudal pontine reticular nucleus^37^,^38^. The most-studied freezing responses are those generated after fear conditioning, in which acoustic stimuli are paired with foot shocks^13^. It is interesting to note that in our study, sound stimuli only induced flight responses; we never observed freezing behaviour in response to our sound stimuli. This observation is in fact consistent with the known function of some components of the identified neural pathway, in particular the ICx to PAGd connection. Previous studies have suggested that the dorsal part of the PAG is involved in organizing flight responses, while freezing responses are primarily mediated by the ventral part of the PAG (PAGv)^34^,^35^, which is not a direct target of the ICx. It is therefore likely that the sound-induced freezing behaviour after fear conditioning involves a different neural circuit, which recruits the PAGv.

In this study, we show that the activation of corticofugal projections to the IC can directly initiate a flight response and that silencing the ACx reduces the sound-induced flight response by nearly half. One parsimonious explanation is that the ACx amplifies the sound-induced flight response via the same neural pathway as it drives the behaviour. The fact that the ICx itself receives ascending auditory information (in particular, from nuclei of the lateral lemniscus)^39^ can explain the result that silencing the ACx does not completely eliminate the sound-induced flight response. Our results suggest that via the corticofugal projections, the ACx may provide evaluative information about the threatening nature of acoustic signals to the ICx, reinforcing the responses in the midbrain. Given that the earliest sound-evoked spike responses occur in L5 among cortical layers^28^, this may allow the ACx to rapidly transform threatening signals to behavioural commands.

In the central auditory system, massive corticofugal feedback projections run in parallel with the ascending projections^20^,^29^. Corticofugal systems are generally thought to be involved in modulating auditory signal processing. For example, they have been shown to act as a positive feedback to augment the auditory responses of tonotopically matched thalamic or collicular neurons^40^,^41^,^42^. Corticofugal systems are also implicated in mediating activity-dependent plasticity in subcortical structures^40^,^41^,^43^. More recently, corticofugal projections to the striatum have been shown to affect the decision-making during auditory discrimination tasks^25^. Our current results reveal a previously unrecognized functional role of corticofugal projections in mediating innate defense behaviours. In particular, our specific optogenetic manipulations strongly suggest that it is the ICx, not the ICC that directly mediates the sound-triggered flight behaviour. This finding indicates that ICC and ICx are functionally differentiable, and it fits with the anatomical suggestion that the ICx may be involved in sensory integration and sensory-motor interaction^29^,^30^,^33^.

Taken together, the neural pathway from the ACx to the ICx and then to the PAG highlights a more active behavioural role of corticofugal projections. By bridging cortical processing of sensory inputs and defensive motor outputs, this pathway may serve not only to amplify innate behavioural responses, but also to provide a route for the top–down control of defensive behaviours in general. Since corticofugal projections are prevalent across sensory modalities, it is possible that direct sensory cortical control of defensive behaviours through corticofugal projections is a general strategy exploited by innate defense circuits.

## Methods

### Head-fixed animal preparation

All the experimental procedures used in this study were approved by the Animal Care and Use Committee at the University of Southern California. Male and female C57BL/6J mice aged 6–12 weeks were used in this study. Mice were housed with reversed light–dark cycles with light on from 21:00 hours to 9:00 hours. Flying saucer pet exercise wheels were placed in their home cages. One week before the behavioural tests, the animals were prepared in a similar way as previously described^17^,^18^,^19^. The mouse was anaesthetized with isoflurane (1.5% by volume) and a screw for head fixation was mounted on top of the skull with dental cement. A ring-like adaptor was glued into the ear that would be contralateral to the sound source for the later ear plugging in some experiments as to avoid the complication of bilateral hearing. Afterwards 0.1 mg kg^−1^ buprenorphine were injected subcutaneously into the mice before they were returned to home cages.

During the recovery period, the mice were trained to be accustomed to the head fixation on the recording setup. To fix the head, the head screw was tightly fit into a metal post. The animals were allowed to run freely on a flat plate rotating smoothly around its center. One day before electrophysiological recordings or behavioural tests that did not require the implantation of cannula, the mouse was anaesthetized with isoflurane and a craniotomy was made over the ACx, IC or SC region accordingly.

The following recordings and tests were all performed in a sound-attenuation booth (Acoustic Systems). The ear contralateral to the sound source was plugged for experiments except those where optogenetic activation was performed. Plugging the ear did not reduce the sound-triggered running speed compared with conditions when both the ears were left open (see Supplementary Fig. 1a). Individual recording sessions lasted for no more than 2 h. The animal was given drops of 5% sucrose through a pipette every hour. During the test, behaviours of the animal were recorded with a video camera. The rotating speed of the plate was detected with an optical sensor and recorded in real time. The speed of the animal was analysed both online and offline. Some animals were tested for more than one session, and the two consecutive sessions were separated by at least 1 day.

### Behavioural test in freely moving animals

A box with two chambers was used to test the sound-triggered flight behaviour in freely moving mice. There was one opening connecting the two chambers, allowing the animal to move between the chambers. Animals were allowed to acclimate to the box environment several hours before the behaviour test. A speaker was attached to each chamber. Noise was delivered randomly by one of the speakers. Silent sound was delivered with zero sound output. Noise levels in both the chambers were measured with a ¼-inch free-field microphone connected with type 2669-L preamplifier (Brüel & Kjær 4939-L-002). There was ∼10 dB difference between the sound source and the far end of the second chamber. Two cameras were attached to the top of the chambers to monitor the location of the animal. Moving speed was calculated from video images.

### *In vivo* recordings in awake animals

Loose-patch, multiunit and LFC recordings were performed as previously described^18^,^19^, with a patch pipette filled with an artificial cerebral spinal fluid (ACSF; 126 mM NaCl, 2.5 mM KCl, 1.25 mM Na_2_PO4, 26 mM NaHCO_3_, 1 mM MgCl_2_, 2 mM CaCl_2_ and 10 mM glucose). Signals were recorded with an Axopatch 200B amplifier (Molecular Devices) under voltage-clamp mode, with a command voltage applied to adjust the baseline current to near zero. Loose-patch recording signals were filtered with a 100–5,000 Hz band-pass filter. LFC signals were low-pass filtered at 300 Hz. The recording sites were marked. The depths of the recorded neurons were determined based on the micromanipulator reading. Recording was performed in a similar way as the whole-cell recording, except that a loose seal (0.1–0.5 GΩ) was made on the cell body, allowing spikes only from the patched cell to be recorded. With large pipette openings (impedance<6 MΩ), no cortical fast-spiking neurons were ever recorded, suggesting that these recording parameters imposed a strong sampling bias towards pyramidal neurons, which have larger cell bodies and more extensive dendritic fields than inhibitory neurons. This bias was also shown in our previous studies^21^,^44^,^45^,^46^,^47^,^48^.

Recording in the ACx: auditory cortical region was pre-mapped with extracellular recordings and the primary ACx was identified by its tonotopic representation of characteristic frequencies in a caudal-to-rostral (low to high frequency) gradient, relatively sharp spike tonal receptive fields (TRFs) and short onset latencies, as we previously described^19^,^21^. Recordings from L5 neurons were mostly based on the travel distance of the pipette beneath the pial surface, and verified by histology in some experiments with fluorescence dextran marking, as we described previously^19^,^21^,^44^,^45^. In addition, it is known that ChR2 in the Rpb4-Cre line would be expressed specifically in L5 neurons. Recordings from neurons (presumably excitatory) at the expected depths confirmed that they could respond to LED light illumination (for example, Fig. 2n).

Recording in the MGBv: We first mapped the auditory thalamus by recording multiunit spikes with a parylene-coated tungsten electrode (2 MΩ, FHC). Mapping was performed in a three-dimensional manner by systematically varying the depth and the *x*–*y* coordinates of the electrode that penetrated the primary auditory cortical surface with an approximately right angle. We distinguished the MGBv from other auditory thalamic divisions based on its tonotopic frequency representation, relatively sharp spike TRFs and short onset latencies^21^. Afterwards, cell-attached recordings were made around the central region of the MGBv (∼ 2.4∼2.6 mm below the auditory cortical surface).

Recording in the IC: The IC area was first mapped by recording multiunit spikes with a tungsten electrode. The ICC region was identified based on short response latencies (6–10 ms for noise responses), sharply tuned TRFs as well as a dorsal-to-ventral gradient of characteristic frequencies (from low to high), as described in our previous study^18^.

Recording in other midbrain structures: The recordings from PAGd and SC were carried out with glass micro-electrodes. The recording pipette was filled with 0.1 mM fluorescence dextran. After recording, the dye was pressure-injected to the recording area and the brain was fixed and sectioned to verify the location of the recording.

### Viral injection

Viral injections were carried out as we previously described^21^. Adult Rbp4-Cre (MMRRC), Pvalb-Cre × Ai14 tdTomato reporter (The Jackson Laboratory) and wild-type C57BL/6J (The Jackson Laboratory) mice were anaesthetized with 1.5% isoflurane. A small cut was made on the skin covering the left ACx or IC and the muscles were removed. Two ∼0.2-mm craniotomies were made in the ACx or IC region. The following adeno-associated viruses (AAVs) encoding ChR2, ArchT or GFP were used depending on the purpose of experiments and strain of mice: AAV1.CaMKIIa.hChR2(H134R)-eYFP.WPRE.hGH (UPenn vector core, Addgene 26969), AAV9.EF1α.DIO.hChR2(H134R)-EYFP.WPRE.hGH (UPenn vector core, Addgene 20298), AAV1-CAG-ArchT-GFP (UNC vector core, Addgene 29777), AAV1-CAG-FLEX-ArchT-GFP (UNC vector core, Addgene 28307) and AAV1.CamKII0.4.eGFP.WPRE.rBG (UPenn vector core). The viruses were delivered using a beveled glass micropipette (tip diameter: ∼40 μm) attached to a microsyringe pump (World Precision Instruments). For each injection, 100 nl of virus was injected at a rate of 20 nl min^−1^. Right after each injection, the pipette was allowed to rest for 4 min before withdrawal. The scalp was then sutured. Following the surgery, 0.1 mg kg^−1^ buprenorphine was injected subcutaneously before returning the animals back to their home cages. Mice were allowed to recover for at least 3 weeks.

### Retrograde tracer injection and imaging

For retrograde tracer injections into IC and SC, 80 nl of fluorescently conjugated Cholera toxin (CTb 555 or 647, 0.25%; Invitrogen) was injected into each location through a pulled glass micropipette using the pressure injection method^49^. Following 5–7 days, animals were deeply anaesthetized and transcardially perfused with 4% paraformaldehyde. Brain tissue was sliced into 150-μm sections using a vibratome and sections were mounted onto glass slides and imaged using a confocal microscope.

### Sound and LED stimulation

Sound stimulation, LED stimulation and data acquisition software was custom-developed in LabVIEW (National Instruments). For behavioural tests with varying intensities, broadband white noise (1–64 kHz) at nine intensities (10–90 dB SPL spaced at 10 dB) was calibrated and delivered pseudo-randomly through a danish speaker (Scan-speaker D2905). In all other cases, the noise intensity was fixed at 80 dB SPL. The duration of noise was 5 s and the inter-stimulus interval was 30 s. The stimulus was repeated 50–80 times to generate an averaged time-dependent speed profile. Note that in quiet conditions animals usually spent only a small fraction of time running, resulting in a low baseline speed. To activate ChR2, a 5-s long train of 10 Hz blue LED light pulses (duration of each pulse was 50 ms) was delivered. The intensity of LED was 12 mW (measured at the tip of the fibre). To activate ArchT, a 5-s long train of 10 Hz green LED light pulses (duration of each pulse was 50 ms, 12 mW) was delivered. The sequence of sound stimulation alone and sound-plus-light combination was randomized.

### Optogenetic preparation

To activate or silence ICx, an optic fibre (400 μm, Thorlabs) connecting to a blue or green LED source (470 nm and 530 nm, respectively, Thorlabs) was positioned close to the surface of ICx. The tip of the optic fibre was covered by agar stained with black pigments to prevent light leakage. Similarly, to activate the ACx or to silence it by activating PV neurons, an optic fibre connecting a blue LED source was placed on the surface of the ACx. The LED-induced behavioural responses could not be attributed to the potential activation of the visual system, since the same optic fibre, when placed on the uninfected ACx, failed to induce changes in running speed (Supplementary Fig. 2e,f). To stimulate deeper structures such as ICC, SC and PAGd, optic fibre patch cord (200 or 400 μm Core, 0.22 or 0.39 NA (numerical aperture) respectively; Thorlabs) connecting the LED light source to the implanted cannula was secured by a hard plastic sleeve (Thorlabs). The implantation was made in the mouse anaesthetized with isoflurane (1.5%) and mounted to the head-fix apparatus. A craniotomy over the target area (ICC, PAGd or ILSC/DLSC) was made. The cannula was lowered with a motion controller (Siskiyou) to the desired depth (ICC: 700 μm; PAGd: 1200 μm; ILSC/DLSC: 800 μm) according to the coordinates in mouse brain atlas. The cannula was then secured on the skull by dental acrylic. All implants were made in left hemisphere. The animals were allowed to rest for at least 2 days before behavioural test sessions. After each experiment, the brain was sectioned and imaged with a fluorescence microscope to confirm the expression of ChR2-EYFP or ArchT-GFP, as well as the location of the implanted cannula. To ensure the specificity of the optogenetic stimulation, no ChR2-expressing fibres and structures other than the targeted structure should be present in the light pathway from the end of the optic fibre. The axis of the light pathway was the same as the central axis of the optic fibre, and the illumination angle was determined by the fibre’s NA value. This rule applied for all of our experiments and fluorescence expression in the brain sections was examined after each experiment to confirm it.

### Silencing of brain structures

We used two methods to silence neural activity. In the first method, fluorescent muscimol or muscimol (1.5 mM, Life Technologies), an agonist of GABA_A_ receptors, was used to silence a targeted brain region^50^. The muscimol solution (dissolved in ACSF containing fast green) was injected via a glass micropipette with a tip opening of ∼2 μm in diameter. To silence the ACx, the pipette was inserted to a depth of 400 μm below the cortical surface. For IC silencing, the pipette was inserted to a depth of 600 μm below the surface. Solutions were injected under a pressure of 2–3 p.s.i. for 5 min. The injected volume was estimated to be around 100–150 nl, as measured in mineral oil. The spread of muscimol^51^ could be precisely measured by fluorescent imaging. Two hours after injection, the animals were transcardially perfused with 4% paraformaldehyde in phosphate-buffered saline. Coronal brain sections (100 μm) were made with a vibratome (Leica Microsystems) and imaged on a confocal microscope (Olympus). The lateral spread of fluorescent muscimol was ∼1 mm.

For optogenetic silencing, AAV9.EF1α.DIO.hChR2(H134R)-EYFP.WPRE.hGH was injected into the ACx of Pvalb-Cre; Ai14 mice^21^,^22^ or AAV1-CAG-FLEX-ArchT-GFP was injected into the ACx of Rpb4-Cre mice. All the injections targeted the primary auditory cortical region, but the viral infection was often observed also in the regions surrounding the primary ACx. In each animal, injections were made in two sites at two depths (300 and 600 μm below the surface). After a recovery period of 4–8 weeks, blue LED light was applied to the cortical surface to activate PV neurons, which in turn silenced cortical excitatory neurons^21^,^22^. As we previously described^21^,^22^, the expression of hChR2(H134R)-EYFP in each injected mouse was examined with a fluorescence microscope before and after the experiments. We found that in all the animals, ChR2 was specifically expressed in PV neurons and the efficiency of the AAV transduction was high (>80%, see Supplementary Fig. 8), consistent with previous reports^21^,^22^.

### Slice preparation and recording

Acute brain slices were prepared from viral injected mice after behaviour tests. Following the urethane anaesthesia, the animal was decapitated and the brain was rapidly removed and immersed in an ice-cold dissection buffer (composition: 60 mM NaCl, 3 mM KCl, 1.25 mM NaH_2_PO4, 25 mM NaHCO_3_, 115 mM sucrose, 10 mM glucose, 7 mM MgCl_2_, 0.5 mM CaCl_2_; saturated with 95% O_2_ and 5% CO_2_; pH=7.4). Brain slices of 350-μm thickness containing the IC or SC/PAG regions were cut in a coronal plane using a vibrating microtome (Leica VT1000s). Slices were allowed to recover for 30 min in a submersion chamber filled with the warmed (35 °C) ACSF and then to cool gradually to the room temperature until recording. The spatial expression pattern of ChR2-EYFP or ArchT-GFP in each slice was examined under a fluorescence microscope before recording. Only slices with the correct location for expression were used for further recording. Cells were visualized with IR-DIC and fluorescence microscopy (Olympus BX51 WI) for specific targeting of infected neurons. Patch pipettes (Kimax) with ∼4–5-MΩ impedance were used for whole-cell recordings. Recording pipettes contained: 130 mM K-gluconate, 4 mM KCl, 2 mM NaCl, 10 mM HEPES, 0.2 mM EGTA, 4 mM ATP, 0.3 mM GTP and 14 mM phosphocreatine (pH, 7.25; 290 mOsm). Signals were recorded with an Axopatch 200B amplifier (Molecular Devices) under voltage-clamp mode at a holding voltage of −70 mV, filtered at 2 kHz and sampled at 10 kHz. 1 μM TTX and 1 mM 4-aminopyridine was added to the external solution to record only monosynaptic responses^15^,^31^.

### Photostimulation in slice recording

A mercury Arc lamp was used as the light source. The light was collimated and coupled to the microscope’s epifluorescence pathway and was passed through blue or green filters. A calibrated aperture placed at the conjugated focal plane of the imaged slice was used to control the size of the illumination area. A train of 10 Hz blue light pulses (duration of each pulse was 50 ms) was delivered to test the activation of ChR2-expressing neurons. Similarly, a long pulse of green light (duratio*n*=1 s) was applied to test the inhibition of ArchT-expressing neurons.

### Data analysis

We performed data analysis with custom-developed software (MATLAB, MathWorks). The data were first pooled together for a randomized batch processing and then were categorized according to experimental conditions. The average time-dependent speed profile was smoothed with a smooth function (rloess) in MATLAB. The speed-intensity curve was fitted with a hyperbolic ratio function:




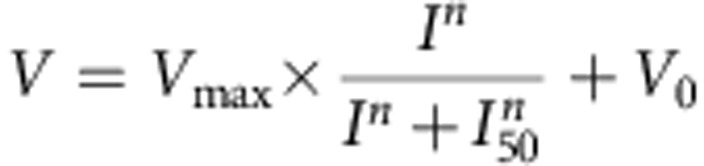




*V*_max_ is the maximum speed and *V*_0_ is the baseline speed.

### Statistics

Shapiro–Wilk test was first applied to examine whether samples had a normal distribution. In the case of a normal distribution, *t*-test or analysis of variance test was applied. Otherwise, a non-parametric test (Wilcoxon signed-rank test in this study) was applied. Data were presented as mean±s.d. if not otherwise specified.

## Additional information

**How to cite this article:** Xiong, X. R. *et al*. Auditory cortex controls sound-driven innate defense behaviour through corticofugal projections to inferior colliculus. *Nat. Commun.* 6:7224 doi: 10.1038/ncomms8224 (2015).

## Supplementary Material

Supplementary Information Supplementary Figures 1-8

Supplementary Movie 1 Freely moving animal in response to noise

Supplementary Movie 2 Head-fixed animal in response to noise

Supplementary Movie 3 Optogenetic activation of corticofugal terminals i k IC cortex

Supplementary Movie 4 Inactivation of corticofugal terminals in IC cortex
