# Supplementary material for: Auditory cortex controls sound-driven innate defense behaviour through corticofugal projections to inferior colliculus
Source: Nat Commun. 2015 Jun 11;6:7224. doi: 10.1038/ncomms8224 (PMC4467028; doi:10.1038/ncomms8224)
Supplement: Supplementary Information — Supplementary Figures 1-8 [file ncomms8224-s1.pdf]

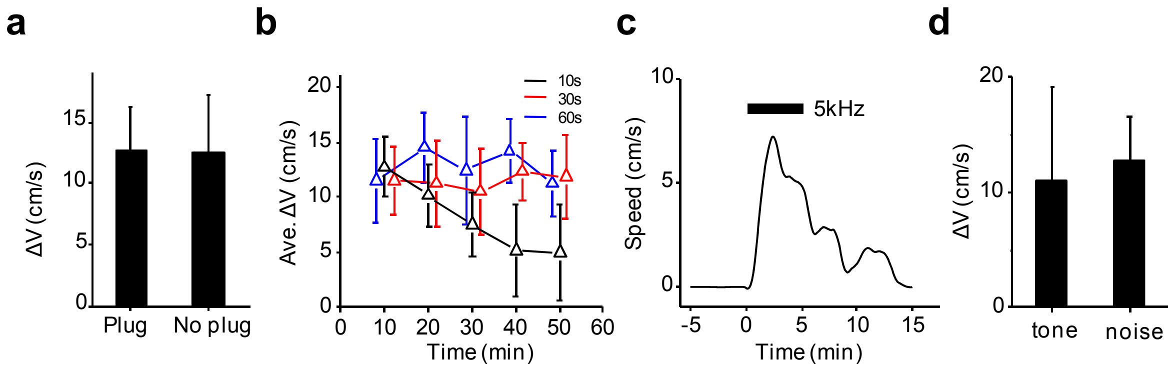

Supplementary Fig. 1. Control experiments in wild-type animals.
a, Similar running responses were evoked when the ear contralateral to the speaker was plugged (n = 35 animals) and when the two ears were left open (n = 25). Error bar = s.d.. No difference was detected. P =0.40, two sample t test.
b,Test of adaptation of the flight response to repeated sound stimulation at different inter-stimulus intervals. Maximum speed induced by sound was averaged for every 10 minutes. Error bar = SD. A two-way ANOVA test showed that the 10s group is significantly different from 30s and 60s groups, P<0.001. N=10 animals.
c, Average speed trace in response to a pure tone (5 kHz; 80 dB SPL) for an example animal.
d, Comparison of induced speeds under tone and noise stimulation.

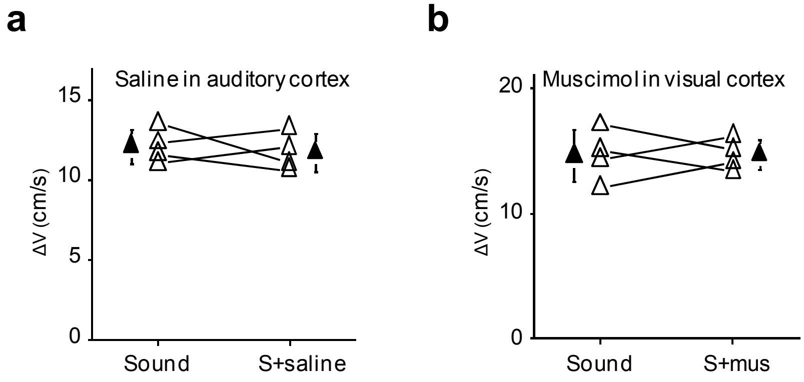

Supplementary Fig. 2. Control experiments for Fig. 2d-h.
a, Noise-induced running speed before and after (S+saline) injection of 150 nl saline into the A1 (n = 4 mice). Data points for the same animal are connected with a line.
b, Noise-induced running speed before and after (S+mus) injection of 150 nl muscimol into the visual cortex (n = 4 mice).

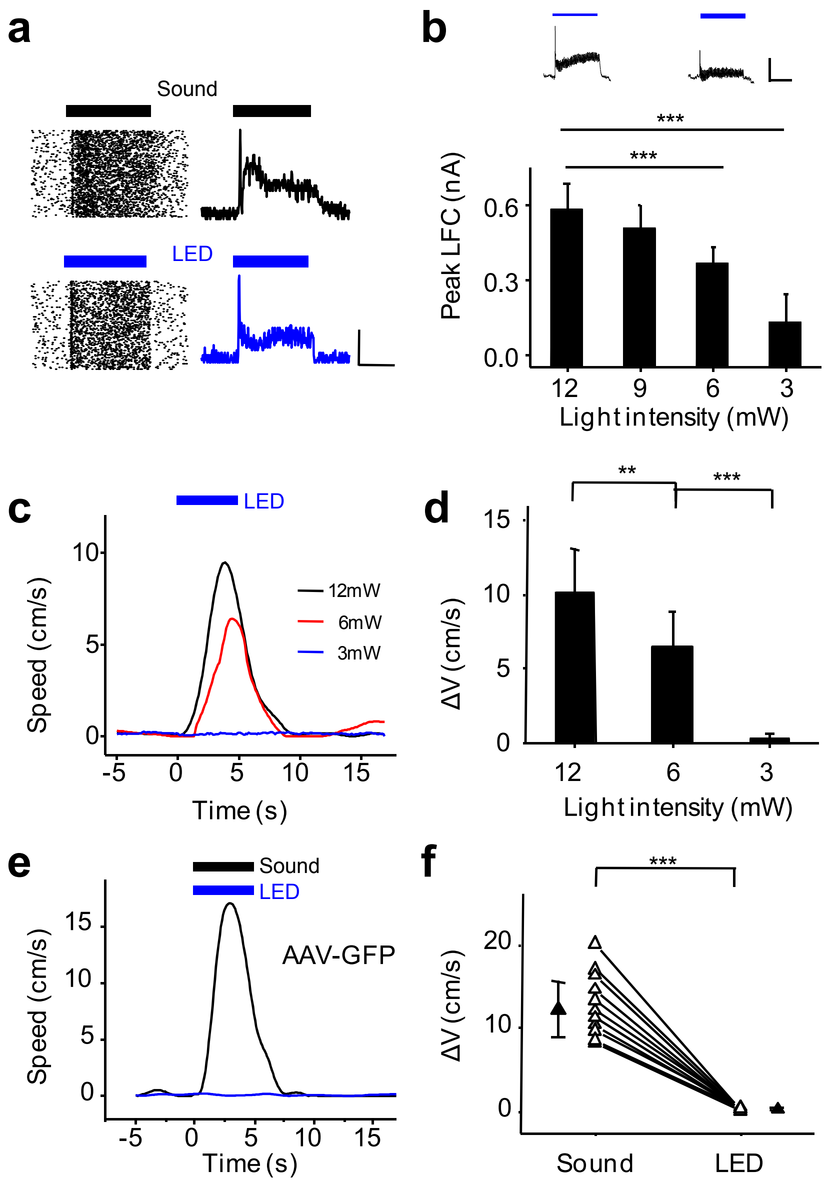

Supplementary Fig. 3. Control LED stimulation experiments.

a, Example rastor plots and PSTHs of multiunit responses evoked by noise (upper) and LED (lower) stimulation for 100 repetitions. The responses were recorded in L5 of the ACx. Scale: 15 spikes, 500 ms.

b, Peak amplitude of local field current (LFC) in respond to LED stimulation as a function of LED light intensity, recorded in L5 of the ACx of a Rbp4-Cre mouse injected with AAV-DIO-ChR2 . Error bar = s.d. Top inset, example traces of recorded LFCs at 12 mW and 6 mW. Scale: 400 pA, 1s. Blue bar marks the duration of LED stimulation. ***, P<0.001, one-way ANOVA and post hoc test. N=20 sites from 5 animals.

c, Average speed traces in response to LED illumination at three LED intensities for the same animal.

d, Summary of average maximum speeds induced by blue LED illumination. **, P < 0.01, ***, P<0.001, one-way ANOVA and post hoc test, N = 9 animals. Error bar = s.d.

e, Average speed traces in response to sound and blue LED illumination on the auditory cortex for a representative wild-type mouse injected with AAV-GFP.

f, Summary of average maximum speeds induced by sound and blue LED illumination in wild-type mice injected with AAV-GFP. ***, P < 0.001, paired t test. N = 11 animals. Error bar = s.d.

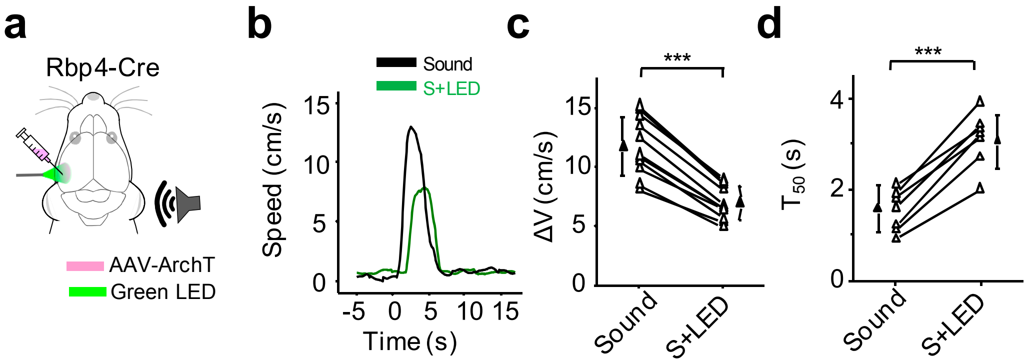

Supplementary Fig. 4. Optical silencing of L5 neurons in Rbp4-cre mice.
a, AAV-ArchT was injected into the ACx of the Rbp4-Cre mouse. Weeks later green LED light was applied to the ACx on the same side, which was contralateral to the ear exposed to noise.
b, Speed trace of an example animal in response to noise only (black) and noise plus green LED illumination (green).
c, Summary of average maximum induced speeds by noise stimulation without and with green LED illumination. ***, $P < 0.001$, paired t test. N = 5 animals and 11 trials.
d, Summary of T_{50} for the same group of animals. ***, $P < 0.001$, paired t test.

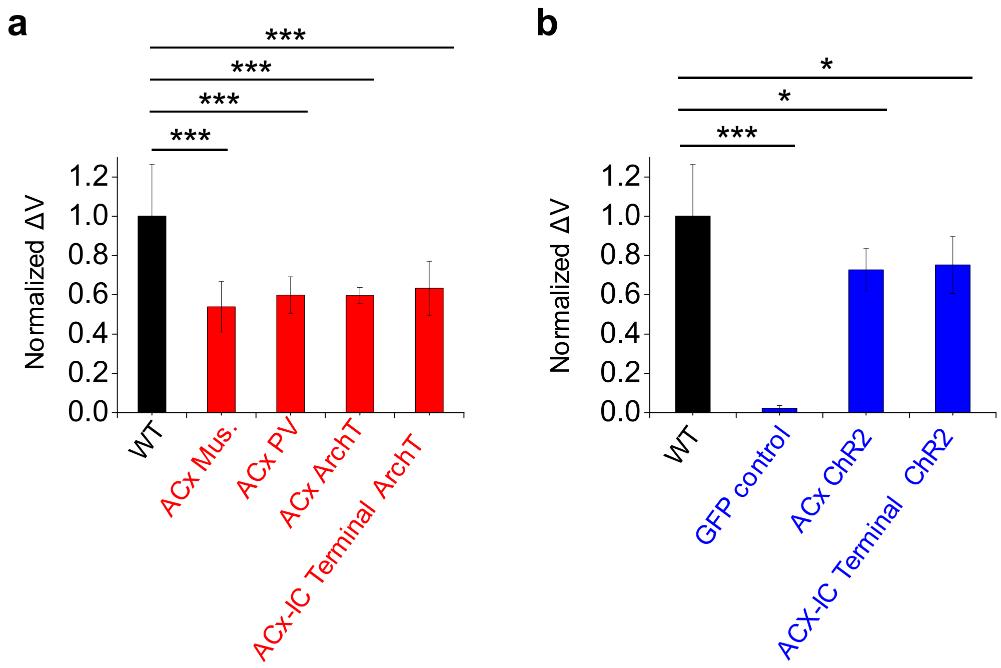

Supplementary Fig. 5. Comparisons of various inactivation and activation manipulations of components of the corticofugal pathway from the Acx to the IC Cortex.

a, Inactivation manipulations. All responses were normalized by the same animal's response to noise only. ***, P<0.001, One way ANOVA post hoc test.

b, Activation manipulations. All responses were normalized by the same animal's response to noise only. ***, P<0.001, One way ANOVA. *, P<0.05, One way ANOVA post hoc test.

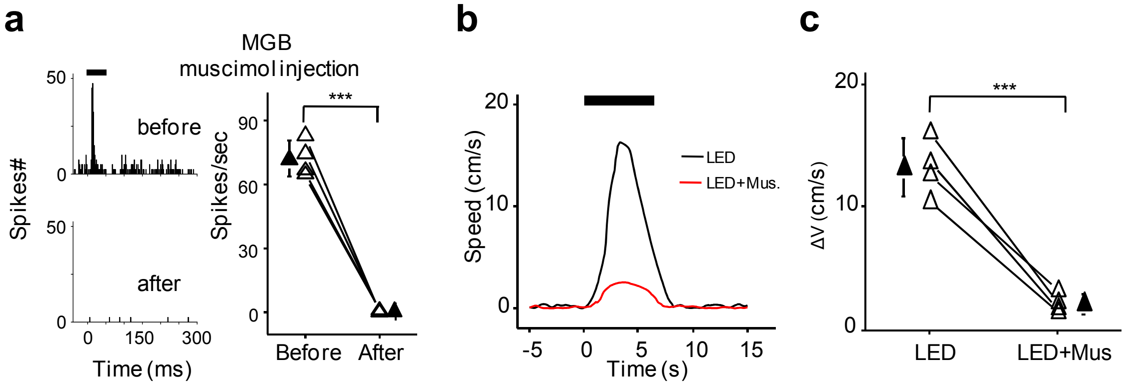

Supplementary Fig. 6. Optical activation of the ICC and silencing of the MGB, a control experiment related to Fig. 4i-l.

Experimental condition was similar as in Fig. 4i-l: AAV-ChR2 was injected into the ICC. Weeks later, the blue LED was applied to the IC surface. The location of MGBv was mapped with multiunit recordings in response to tone stimuli. The MGB later was silenced with injection of muscimol.

a, Left, PSTHs for multiunit spike responses of one recording site in the pre-mapped MGBv to noise stimulation (60 dB SPL, marked by the thick bar) before (upper) and after (lower) the muscimol injection into the MGB. Right, summary of average spike rates evoked by noise before and after the muscimol injection. $P < 0.001$, paired t test. N = 4 recording sites in 4 animals.

b, Speed trace of an example animal in response to blue LED illumination before (black) and after (red) injecting muscimol into the MGB.

c, Summary of average maximum induced speeds to LED illumination before and after injecting muscimol into the MGB. $P < 0.001$, paired t test. N = 4 animals.

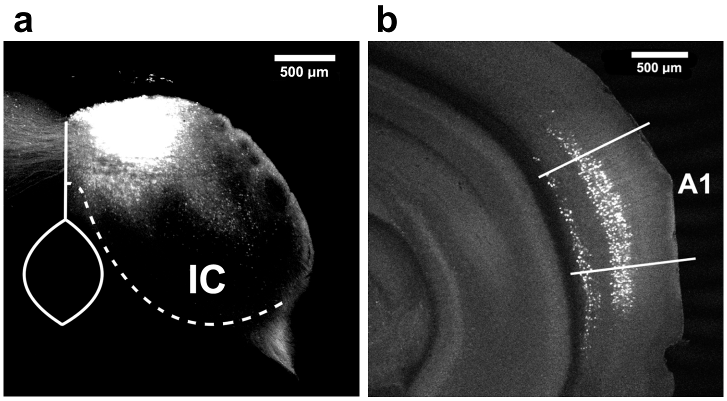

Supplementary Fig. 7. Retrograde labeling of IC-projecting auditory cortical neurons.
a, Image of CTb injection site in the inferior colliculus.
b, Image of retrogradely labeled neurons in the cortex. Note that there are more labeled neurons in the primary than secondary auditory cortical regions. The boundaries of A1 are marked.

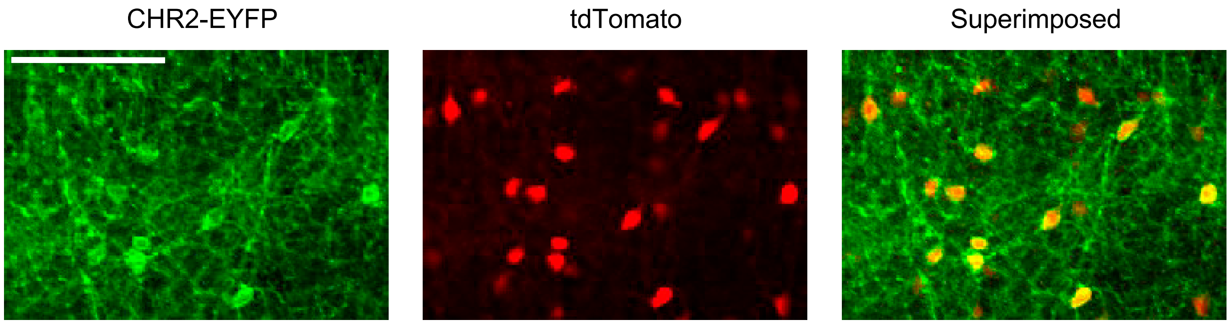

Supplementary Fig. 8. Specificity of expression of ChR2 in PV neurons.
Images were obtained four weeks after injection of AAV-DIO-ChR2-EYFP in a PV-Cre; Ai14 mouse. Left, ChR2-EYFP signal. Middle, tdTomato signal. Right, superimposed image. Scale bar, 100μm.
